# Progress and Challenges: Implementation of the UK Antimicrobial Resistance National Action Plan 2019–2024 within the Beef Cattle Sub-Sector

**DOI:** 10.3390/antibiotics13090839

**Published:** 2024-09-04

**Authors:** Houda Bennani, Louise Whatford, Jessica Myers, Nicholas Mays, Rebecca Glover, Barbara Häsler

**Affiliations:** 1 Veterinary Epidemiology, Economics and Public Health Group, Department of Pathobiology and Population Sciences, Royal Veterinary College, London NW1 0TU, UK; lwhatford@rvc.ac.uk (L.W.); jmyers20@rvc.ac.uk (J.M.); bhaesler@rvc.ac.uk (B.H.); 2 Policy Innovation and Evaluation Research Unit, Department of Health Services and Policy, London School of Hygiene & Tropical Medicine, London WC1H 9SH, UK; nicholas.mays@lshtm.ac.uk (N.M.); rebecca.glover@lshtm.ac.uk (R.G.)

**Keywords:** antimicrobial use, antimicrobial resistance, beef, AMR policy, animal health and welfare

## Abstract

The five-year UK antimicrobial resistance (AMR) National Action Plan (NAP) was published in 2019 focusing on reducing the need for, and unintentional exposure to antimicrobials (AMs); optimising the use of AMs; and investing in innovation, supply and access of AMs. This study aimed to evaluate the progress made in the beef cattle sub-sector in addressing specific NAP commitments related to improving animal health and welfare and responsible antimicrobial use (AMU). A thematic analysis was conducted of 21 semi-structured interviews with stakeholders from government organisations, farms, veterinary practices, levy boards and livestock associations. The findings indicate substantial progress, with various initiatives implemented targeting data collection, farmer and veterinarian engagement, and herd health planning. However, there remain a number of challenges and barriers that need to be addressed in order to assess the impacts of these initiatives, such as the availability of AMU and AMR data. Ensuring the adequacy of resources was found to be critical for the sustainability of effective initiatives, considering competing demands on people’s time. Additionally, the importance of other outcomes from these initiatives such as developing and strengthening the farmer–veterinarian relationship should not be underestimated since it is fundamental to successfully addressing issues such as AMR.

## 1. Introduction

Antimicrobials (AMs) are active substances of synthetic or natural origin that kill or inhibit the growth of microorganisms (bacteria, viruses, fungi and parasites); they are critical for fighting diseases in humans, animals and plants [1]. In animals, AMs are also used in a non-therapeutic way for disease prophylaxis (i.e., AMs administered to a herd or a flock at risk of disease), which is an increasingly discouraged practice globally; and for metaphylactic purposes (i.e., AMs administered to healthy animals in a herd or flock of animals where some have already shown clinical signs of infection) [2]. They are also used as growth promoters (GPs) in subtherapeutic dosages to increase productivity [3], although this practice was banned in 2006 in the European Union (EU) (including the UK) as a response to increasing concerns about the effect of this type of use on antimicrobial resistance (AMR) [4]. Resistant organisms and resistant genes are present in humans, animals, food and the environment, and can be transmitted between them through various pathways [5,6,7]. The main driver of AMR is the use of AMs, and overuse and misuse of AMs have greatly accelerated the pace at which AMR develops and spreads [8,9]. Hence, responsible use of AMs in humans, animals, plants and the environment is key to tackling this growing challenge. Other factors also contribute to the emergence and spread of AMR such as poor infection and disease prevention and control; inadequate access to clean water, sanitation, and hygiene; lack of equitable access to affordable and quality assured AMs and vaccines; and discharge from healthcare facilities, pharmaceutical manufacturing and farms [10].

The UK government published its five-year AMR National Action Plan (NAP) in 2019 based on One Health (OH) principles and focusing on three key areas for tackling AMR: (1) reducing the need for, and unintended exposure to AMs; (2) optimizing the use of AMs; and (3) investing in innovation, supply and access [11]. The Policy Innovation and Evaluation Research Unit (PIRU) at the London School of Hygiene and Tropical Medicine, was commissioned to undertake an evaluation of the implementation of the NAP in the context of the pandemic, in partnership with the Royal Veterinary College and the Centre for Ecology and Hydrology, to contribute to the update of the NAP. This article focuses on the part of the evaluation investigating progress made in the beef cattle sub-sector since the launch of the NAP in 2019. Beef cattle was chosen as a case study, because of the potential role of this sub-sector for AMU and AMR due to its size (1.46 million beef cattle in 2022) [12], its considerable food output (906,400 tonnes of beef and veal meat in 2021) [13], the connection with the environment through extensive production, the scarcity of AM usage data and the relatively (compared to other livestock) low frequency of veterinary involvement in production [14,15]. Data from 2020 showed that the total UK livestock output had a value of over GBP 15 billion, with the largest proportion (nearly 30%) attributed to the dairy sub-sector followed by the beef cattle sub-sector [16]. The UK is approximately 75% self-sufficient in beef and is one of the most significant beef producers in Europe [17].

Beef cattle are primarily raised for meat production with the goal of producing high-quality meat for human consumption and are kept in all parts of the UK in different production systems. Beef derives from two main sources: the suckler herd (bred for meat); and dairy herd production systems [18,19]. In the suckler industry, calves are reared by their mothers until they are weaned at around 7–10 months of age and then fattened ready for slaughter around 2 years of age and approximately 550–650 kg in weight (finished) [18,19]. Cattle supplied for beef production from the dairy herd are either calves born to dairy cows or cows at the end of their dairy life [20]. Calves are usually sold by dairy farmers to beef farmers who will rear them until ready for slaughter (finishing). There has been a gradual increase in using dairy calves for beef, to minimise waste in the systems, in particular for male calves, with the Agriculture and Horticulture Development Board (AHDB) estimating an increase in beef-registered calves from dairy dams from 25% in 2019 to 35% in 2021, highlighting the importance of this stream of beef [21]. 

The UK livestock sector, through the Responsible Use of Medicines in Agriculture (RUMA) alliance, has been working in collaboration with the Government’s Veterinary Medicines Directorate (VMD) to promote the responsible use of AMs [22]. RUMA is an independent non-profit group, which was established in 1997 with the goal of promoting the highest standards of food safety and animal health and welfare in the UK livestock industry [14]. The RUMA Targets Task Force (TTF) was formed in 2016 to develop specific targets for the reduction in antibiotic use (ABU) and comprised a specialist veterinarian and leading farmer for each of the sub-sectors of beef, dairy, eggs, fish, gamebirds, pigs, poultry meat and sheep [14,15,22]. In addition, the British Veterinary Association (BVA), the Red Tractor Farm Assurance scheme and the National Office for Animal Health (NOAH) also attended as observers [14]. The first set of targets was for the period 2017–2020 and the second one covered the period 2021–2024 [15,22]. The TTF took a holistic approach to antibiotic stewardship and covered not only a reduction in ABU but also the development of improved data collection systems for antibiotic usage, improved husbandry and biosecurity practices, and training on stewardship for farmers and veterinarians [14,15,22]. As a result of this collaboration between industry and government, antibiotic sales for food-producing animals decreased by 59% between 2014 and 2022 to reach 25.7 mg/PCU (milligrams per population correction unit), and over the same period, the use of Highest-Priority Critically Important Antimicrobials (HP-CIAs) reduced by 82% to 0.12 mg/PCU in 2022 [23]. 

The aim of this study was to investigate the progress made in the beef cattle sub-sector in addressing the following commitments stated in the NAP [11] and updated in the 2022 addendum [24]:

- Implement plans with the veterinary profession and livestock industry to improve animal health and address endemic disease issues through disease control schemes, veterinary advice and health planning, and tools for promoting knowledge transfer (such as guidance, training and communication).
- Incentivise regular monitored animal health and welfare review as a key strategy for infection prevention and control in farmed animal enterprises. Monitor the effectiveness of animal health and welfare interventions to learn and disseminate best practices at regional and national levels.
- Encourage greater uptake of available vaccines.
- Work with industry and the veterinary profession to improve our understanding of available disease data. Consider how to expand this and share at farm, regional and species levels as well as nationally. Use in tandem each country’s multispecies databases to improve disease surveillance and reduce antibiotic use.
- Work collaboratively across, for example, UK government administrations, the veterinary profession and the agriculture industry to develop appropriate training, guidance and other communications for those who are (or influence or are training to be) antimicrobial users and prescribers to encourage the uptake of recommended practices. And evaluate their impact.
- Improve the accuracy, availability and coverage across the UK of antibiotic use data in the main livestock sectors. This will include working collaboratively across UK government administrations, the veterinary profession and agriculture industry to implement sector targets by the end of 2024.

## 2. Results

### 2.1. Awareness of the NAP and Views on ABU in the Beef Sub-Sector 

Gauging the extent to which individuals and groups relevant to the NAP are informed about its existence, purpose, and intended outcomes helps to know whether there is general support and buy-in for the plan, whether communication efforts have effectively disseminated information about the plan and whether stakeholders comprehend its goals and find its implementation necessary.

#### 2.1.1. Awareness of the NAP 

There was little awareness of the AMR NAP among veterinarians interviewed, most had heard the term, and they had sound knowledge of the initiatives, but not within the context of the specific NAP commitments. Similarly, farmers interviewed had very little awareness of the NAP and were more aware of the work of RUMA TTF which leads to responsible AMU in FPAs in the UK. RUMA TTF develops voluntarily sector-specific targets, and designs and implements interventions to achieve them [14]. When asked about the influence of the NAP on RUMA’s work, the informant from RUMA explained that it takes the NAP into consideration when developing sector-specific targets. Having a representative from the VMD in the RUMA TTF was also seen to help ensure that the knowledge about the NAP fed into the discussions: “*The second one (AMR NAP 2019–2024), yes, through the VMD’s involvement. But we were aware of it. In our business plan every year, we state that our actions will contribute to the National Action Plan. So, we’re aware of it, and we will—we’re aware of our responsibility to contribute to it. Is it putting pressure on us? Is it driving us, is it motivating us? No, not really, because we are committed to the One Health concept*” (Senior Staff). 

#### 2.1.2. Perception of ABU in Beef Cattle (and Its Evolution) 

In general, interviewees were not very concerned about the volume of antibiotics used in beef cattle. They thought that they were used responsibly and there was a feeling that the use had improved in recent years. Veterinarians argued that they were diligent in avoiding the unnecessary prescribing of antibiotics and were knowledgeable about which ones to prescribe first and which ones to avoid: *“I mean, I wouldn’t say I feel worried or anxious, to be honest, but I would say I have a professional concern that I’m aware that I don’t want to use them anymore than feels necessary. And I want to make sure they’re used appropriately when they’re used and I want to try and help the farmers to manage their farms so that they’re not needed”* (Vet 2). Veterinarians felt motivated to improve their practices and refine their use of antibiotics as their knowledge on the subject expanded. 

Beef farmers interviewed expressed that they did not see ABU as a major issue. They emphasized that their antibiotic treatments are primarily for addressing isolated health issues and that they had changed their practices: “…*I mean 10 or 20 years ago we were reaching for antibiotics a lot more. But we are not now, maybe an odd foul of the foot. This time of year, you have flies quite a bit, I might have to inject one with Engemycin for their eye*” (Farmer 4). When asked why they think this had improved, Farmer 4 said: “*Well instead of reaching for it, we have been encouraged to use less and we’ve learned to use less really. Well for my own part I think I’m fairly responsible really. But having said that from an economic point of view, I did use Alamycin to control the pneumonia*”. Veterinarians observed that farmers had become more aware of the need to reduce antibiotic use and were making efforts to minimize it. Farmers recieve this messaging from external sources such as veterinarians, assurance schemes and livestock associations. As a result, some farmers had made changes in their management to prevent disease and consequently avoid the need to use antibiotics: “*I would say on individual farms, yes, I can think of several calf rearers in particular who have made significant changes to their management which mean they’re not using nearly so many antibiotics to treat calf pneumonia or calf scours*” (Vet 2).

Overall, interviewees perceived ABU in the beef cattle population to be low compared to other livestock sub-sectors. Where concerns were mentioned, they related more to calf-rearing units, where management could be improved such as in relation to their environment, colostrum use and management (e.g., avoiding overstocking of calves in indoor intensive production systems): *“Probably at least in that I worry that there is a bit of usage to cover for poor management of the young stock particularly. So, two conditions that really come to mind are pneumonia and diarrhoea, treating those ones”* (Vet 5). Calf-rearers raise young animals predominantly from the dairy sector [15]. Mixing calves from different sites combined with stress from transport increases the risk of disease transmission at a time when calves are more susceptible to disease [25]. An increase in the use of dairy calves for beef production has been reported in the UK due to the commitments to eliminate euthanasia of calves [26]. A study that investigated antimicrobial practices in beef farmers in England and Wales showed that farms with rearing units were associated with higher AMU [25]. In the second phase of the targets covering the period 2021–2024, RUMA TTF created a separate category for calves under six months of age reared away from the cow to draw attention to this area [15]. 

### 2.2. Initiatives Implemented in the Beef Sub-Sector 

#### 2.2.1. Rationale/Link to NAP Actions or RUMA Targets 

A range of initiatives have been implemented in the beef cattle sub-sector relating to data collection, farmer and veterinarian engagement, and herd health planning. They relate directly to different NAP commitments, as indicated in Table 1. 

#### 2.2.2. The Medicine Hub

The Medicine Hub (MH) is a web-based recording system developed by AHDB, the levy board representing agriculture and horticulture for England, to record ABU data in dairy, beef and sheep production systems. The MH was launched in spring 2021 and data from this hub are expected to be used for reporting nationally. Interviewees regarded the MH as a significant initiative to improve data capture, noting that it demanded substantial effort and commitment from the sector. The target for the beef sub-sector was to collect data from 8000 (10% of total) UK beef herds by 2024. For calves, the target was to collect data from 50% of UK calf-rearing units by 2024 [15]. In the latest RUMA TTF progress report published in November 2023, 2968 datasets had been uploaded to the MH for beef by September 2023 [27]. For calves, no figure was published in this latest report but the one published in November 2022 stated that some of the main specialist calf-rearing companies were supplying data to MH [28]. The data from the 2968 beef enterprises uploaded to the MH, which represents 5% of the total number of beef enterprises in the UK (there are approximately 60,000 in total), were used to calculate a mean antibiotic usage of 4.8 mg/kg and a mean HP-CIA use of 0.01 mg/kg [27]. These figures were calculated using the Cattle Health and Welfare Group (CHAWG) methodology, which uses a denominator based on the overall population of beef cattle which are “at risk” across a range of animal categories and standard weights [29]. This is different from estimates produced with the mg/PCU methodology that uses only slaughtered beef animals as a denominator, which means that total antibiotic use is distributed over a smaller number of animals [23]. 

All veterinarians interviewed were aware of the MH, but most farmers interviewed had not heard of it. All farmers interviewed recorded ABU in their farms using various software programmes. Interviewees reported a slow uptake since the launch of the MH: “*There has been a lot of work on medicine hub to really get that up and running. I still think it’s a bit slower than we want it to be but I think there is progress being made on the medicine hub. So definitely the medicine hub has been one of the key focuses*” (Senior Officer). Various reasons were mentioned for this slow uptake, including the following: (1) the incompatibility of software between the MH and veterinarians’ software; (2) concerns about the time required to upload the data to the system; (3) the heterogeneous nature of the beef, lamb and dairy sub-sectors in the UK and the large number of producers that make unified messaging a challenge; and (4) some veterinarians and farmers in the ruminant sector considering themselves to be low users of antibiotics, so do not necessarily see the importance of engaging and collecting antibiotic use data. 

The following quote illustrates veterinarians’ concern about the lengthy time required to upload the data to the MH, which constitutes a barrier to its uptake: *“At the moment we’ve got a huge amount of admin work being able to pull an excel CSV file out of our practice management software and then alter it to something that medicine hub can deal with and crunch. And at the moment we are paying for that with vet time, there is no funding, farmers don’t want to pay for it, medicine hub don’t want to pay for it. No one wants to pay for that admin time”* (Vet 1). Veterinarians highlighted the need for greater funding and support to improve the technology. The MH also relies on good collection of data at the practice level, e.g., separation by species, which is not consistent among all veterinary practices. 

Despite the slow uptake, interviewees were optimistic about the objectives of the MH, commenting that it would be an excellent data source to understand ABU at the farm level. The MH is a good example of where veterinarian–farmer relationship, trust and communication were deemed crucial to the uptake and delivery of initiatives to monitor and improve ABU. To overcome the slow uptake of the MH, some interviewees suggested focusing initially on veterinary data as an estimate of the quantity of antibiotics used. However, emphasis was placed on the necessity of allocating specific funding for this to be successful: “*There’s lots of discussion at the moment about this, if you were going to use the vet practice model, if you were going to say, right, it’s the vet who must put their medicine sales on to demonstrate what the farms are using. And we know from RVC research and elsewhere that that’s probably the best approximation you will get, even though it’s got inaccuracies in it. If you paid the vets to do that they would resource it and do it, or if you said to the farmers, you have to do this, or, you have to make sure somebody is doing this on your behalf and it was funded*” (Vet 8).

#### 2.2.3. The Welsh Lamb and Beef Producers (WLBP) AMU Calculator 

To collect usage data in the ruminant sector in Wales, another system was developed by the Welsh Lamb and Beef Producers Ltd. (WLBP) called the WLBP AMU Calculator and was launched in 2021. In contrast to the MH that was funded by the livestock industry, the WLBP AMU calculator received funding from the Welsh Government through the Arwain DGC (responsible antimicrobial use) project. Arwain DGC aims to reduce the need to use antibiotics by improving productivity, and animal health and welfare through innovative technology and ‘good practice’. Several activities have been ongoing as part of this project (https://rhaglenni.mentera.cymru/arwaindgc/ (access 1 August 2024)). There was a feeling of pride in the work conducted in Wales to tackle AMR and an emphasis on the importance of collaboration between various stakeholders for the success of addressing NAP commitments: *“You know, generally, I think that it’s been very, very good. I think the key to what we’ve done here in Wales is getting people to work together as a group and as a consortium to deliver the policy goals of the NAP. So, the Arwain DGC group who are delivering it includes vets, farmers, academics, you know, all working together, and I think that’s been the secret of it, really”* (Senior Officer). 

The uptake of the system was higher than the MH. From July 2022, the Farm Assurance Welsh Livestock (FAWL) scheme made it a requirement for farmers to record ABU data electronically in the platform. This process takes place during the annual health and welfare review with the veterinarian. Since half of the beef farms are assured in Wales, this led to data from half of the farms being submitted. In November 2022, 1009 beef farms completed their antibiotic usage in the platform, and this work continued in 2023 [27,30]. It is planned that the data from the Welsh system will feed into the MH but there are still issues related to data protection that need to be resolved. 

#### 2.2.4. Training for Veterinarians and Farmers 

Arwain Vet Cymru (AVC) is a programme that was implemented between 2019 and 2021, which focused on improving antibiotic prescribing in cattle and sheep through a Wales-wide national network of Veterinary Prescribing Champions (VPCs). The AVC initiative included training but also mentoring and building a community of practice. At the end of the project, the Arwain network had at least one trained VPC from close to 90% of farm veterinary practices in Wales: *“Arwain Vet Cymru, and that’s set up, essentially, the network of veterinary prescribing champions, which we led on in Wales. And that was getting vets from every farm animal practice into a group, that would then be the leaders on responsible antibiotic use in their practice. And we focused training and the development and the mentoring on those people, and they cascade it down to their practices. So, that was the first one and that seemed to be very successful”* (Senior Officer). With the support of the Arwain DGC project, the AVC programme led to the formation of two specialist VPC working groups to develop a voluntary Code of Conduct for veterinary prescribing and a set of clinical guidelines for key diseases encountered in farm practice. Both documents were disseminated to the VPC network and are also available to download via the Arwain DGC website (https://rhaglenni.mentera.cymru/arwaindgc/hwb-adnoddau/cyhoeddiadau/ (accessed on 1 August 2024)).

The Farm Vet Champions (FVC) is a UK-wide initiative that was inspired by AVC. The FVC provides online training on antibiotic stewardship to practising farm veterinarians across the UK. The VMD provided funding for the development of the training materials and the SMART (Specific, Measurable, Relevant, and Time-bound) goal programme between May 2021 and May 2022, but there was no funding thereafter. Veterinarians who had completed the training found it useful. They appreciated learning more about how their role in AMR stewardship fitted into the wider context of improving AMU and tackling AMR. Moreover, the importance of this programme in helping with the farmer–vet relationship, and building confidence and transdisciplinary communication skills in veterinarians was highlighted. The farmer–vet relationship and bi-directional trust is a thread running through the study results. It is needed for success in areas such as AMU. Any process that can help strengthen trust should have a positive effect. The fact that it was mentioned that this initiative may also help to provide younger vets with more confidence, perhaps also demonstrates that this relationship takes time to develop: “*In terms of Farm Vet Champions, definitely will help on the farm side, will hopefully give some younger vets, younger members of the practice some confidence to have that discussion with antibiotic use on farm and with farmers*” (Vet 1). There were concerns that training such as FVC will easily be missed or dropped if practices are understaffed and overworked as it will not be a priority amongst all the other tasks to complete. 

When asked about participation in the programme, a veterinarian said that there was good participation at the beginning but then it slowed down: “*So, we had good sign-up when we said, “Here we are, go for it.” Then we launched the smart goals back in May a year ago (in 2022), and we got a little bit of an uplift and people showing a bit of interest. But I would say it’s frustrating because we haven’t snowballed to get mass participation. So, it’s a—and I think that’s vet time, busy, just you know, there’s going to distract—there’s always plenty of distractions. And yeah, people are under pressure, and they’re sort of doing their day job and maybe it’s not top priority*” (Vet 10). The aim was to have 50% of farm veterinary practices participating by 2024 (or 2800 FVCs in 900 veterinary practices) [15]. In September 2023, there were 892 FVCs across the UK, 44 SMART goals were set and 18 teams created [27]. One veterinarian commented on the importance of understanding the challenges of such initiatives and the need to continue promoting it: “*So, I think if you look at the figures on farm vet champion there are high targets but I kind of think it’s a good initiative, it’s an initiative we can carry on promoting it will build. And I think being below targets isn’t necessarily a failure, it’s just a reflection of how difficult it is to change behaviours properly or get people to do something extra when they’re already very busy”* (Vet 8).

Another training initiative for cattle veterinarians is online and in-person training offered by the British Cattle Veterinary Association (BCVA) to support their efforts in managing diseases by taking a responsible approach to the use of antimicrobials. 

Farmer training initiatives were praised for the opportunity they present for engagement between veterinarians and farmers as they learn together how to manage specific health issues on the farm. This again, highlights the importance of these relationships, and the value of being able to learn and talk in both directions. In November 2021, the Red Tractor assurance (the largest farm assurance scheme in Britain) made it a requirement for at least one person in each farm (who is responsible for administering medicines) to undertake an approved medicines training course, which was perceived to be effective: “*I think the one thing that helped a lot was when Red Tractor made it compulsory for staff on the farm to have done a med course. So, you know an awful lot of clients would have done our safe use of meds course which focuses a lot on reducing or refining antibiotic treatments so that’s reassuring*” (Vet 3). However, it has been noted that this would not benefit those who are not Red Tractor-assured farmers and that there are still many who are not, which limits the reach. Quality Meat Scotland (QMS), the levy board representing the red meat industry in Scotland, has a recommendation that for cattle, at least one member of staff responsible for administering medicines should have undertaken training in the administration and handling of medicines [15]. Another aspect mentioned about the farm assurance schemes is that for beef, they are only required for the last 90 days (i.e., finisher farms) and that is not where the highest risk for AMU is. An informant compared the cattle sector to pig and poultry sectors which need to be farm-assured for their whole life. Having a lifetime farm assurance was consulted on in 2016 but it was rejected by beef farmers and farming organisations [31].

#### 2.2.5. Herd Health Plan 

Another initiative that was mentioned was the herd health plan. The objective of this plan is for the farmer and veterinarian to develop a bespoke plan for each farm, and review health and performance indicators annually. The plan addresses specific areas of responsible use of antibiotics and health and welfare that are tailored to the farm. Individual farmers are encouraged to establish their own level of use on-farm using the MH to support discussions with the veterinarian and benchmarking activities. For the beef and calf sub-sectors, the target was to reduce non-compliances annually in Red Tractor Beef & Lamb assurance, FAWL Beef and Lamb Scheme, QMS Cattle and Sheep Assurance Scheme and Northern Ireland (NI) Beef & Lamb Farm Quality Assurance Scheme where there is a requirement to develop a herd health plan with the veterinarian and conduct an annual health and performance review. Non-compliance does not mean that there is no health plan on these farms but that something might be missing. In November 2022, compliance in assured farms was 79% for beef [30]. 

Herd health plans were described as useful in promoting responsible use through a better ability to monitor the use, make changes based on this use and discuss relevant health issues. When asked if they found them helpful, one farmer said: *“Yes, I do because they are [veterinarians] the experts, so in terms of the pneumonia vaccines or anything really, if you don’t speak to them, you would just carry on using the same drugs every year and still get a few odd cases. They recommend what testing to and pinpoint you need to use or not. I don’t want to be using antibiotics every year, if I can avoid it through using the right vaccine I will, saving money, saving time”* (Farmer 6).

Veterinarians appreciate the opportunity that completing the herd health plan gives them to engage with their farmers on management and general health issues beyond a visit purely for treatment. As another example of the importance of having opportunities for bidirectional learning, the herd health plan provides another opportunity for the veterinarian to become more informed as to the idiosyncratic workings of each farm, to allow for better communication and bespoke suggestions, as well as providing a structured way to approach the topics of herd health and management which they believe has been beneficial. Some of the changes in herd management that veterinarians discussed as being beneficial to reducing ABU were implemented in response to the use of the herd health plan. However, it was highlighted that in the beef cattle sub-sector, herd health plans can be underutilized, ignored and not referred to, despite being produced, and may be seen as more of a ‘tick-box’ exercise than a practical useful tool: *“So, yes, they do exist, I could be cynical and say, how many of them are actually active working documents and something that happens on the farm, and how many of them are something that’s dusted off for the farm assurance scheme is always the worry I have. Do all that work and then it doesn’t change anything”* (Vet 5). 

#### 2.2.6. Animal Health and Welfare Pathway (AHWP)

The Animal Health and Welfare Pathway (AHWP) was launched in 2023 and offers financial support to farmers in England to take actions that directly improve the health and welfare of their animals. The support can be through the provision of funding for an Annual Health and Welfare review with a veterinarian (up to GBP 522 for beef cattle), support for disease eradication and control programmes and financial grants to apply for equipment and infrastructure [32]. Veterinarians would carry out diagnostic testing on specified endemic diseases (BVD in beef cattle) and provide advice on management to improve the health and welfare of the animals. The scheme is initially open to those farmers who were previously on the basic payment scheme, which provided financial support to farmers who produce, rear or grow agricultural products including milking, harvesting and breeding, and keeping animals for agriculture and also keeping some land suitable for grazing or cultivation with 5 hectares of eligible land. The basic payment scheme was phased out, with the last payments taking place in 2023. 

Veterinarians had a good understanding of the AHWP, but most farmers had limited knowledge as they mentioned that they had just been made aware of the initiative by their veterinarians. There was a feeling of optimism among veterinarians that the AHWP would offer greater opportunity to engage with farmers and develop a bespoke plan: “*The pathway programme, embraces that concept [herd health plan] and encourages it further. So, it’s providing an incentive for the vet and the farmer to sit down and build a strategy, build a health plan, whatever you want to look—look forward twelve months, where are your hotspots of activity, where are your concerns? Biosecurity, et cetera. Tackle them. The incentive in the pathway, which I think will build up this support/engagement for the health plan generally, is “actually, we can probably help you with some funding to look for some of the diseases” (Senior Staff).* The value of these types of initiatives in building the relationship between veterinarians and farmers as well as allowing more time to discuss farm issues was highlighted. Veterinarians felt that these initiatives would be particularly beneficial to their relationship with beef farmers as they have much less contact with them than dairy farmers: *“I think it probably depends on the farm and I would probably say it will have more of an impact for beef farmers actually because the dairies have a lot of contact with us already”* (Vet 3). 

Some veterinarians expressed concerns that only one visit would not provide the ability to follow up on the areas identified in the review and would not be as effective in driving change as if veterinarians could have the opportunity to check in more regularly with farmers. Others viewed this as part of a step change to improve interaction with farmers, showing them the value of health planning and thereby fostering stronger relationships: *“… if all you do is once a year for a lot of farms that would be inadequate but you’re trying to create interaction with the ones who maybe aren’t doing it. And you’re trying to increase the interaction with the ones who are already doing it and maybe rewarding the ones who are already really, really involved and proactive. So, yes, I just think it’s all part of a cultural shift that we’re trying to encourage*” (Vet 8).

Interviewees were also positive about the grant element of the AHWP and that this would help to improve herd management on farms and would allow the promotion of better housing and biosecurity. In a recent research brief that explored the opportunities and risks of the AHWP, it was reported that overall stakeholders from the beef ad sheep sectors were positive about the AHWP elements and goals, but critical of the amount of funding available [33]. A similar initiative in Wales, the Sustainable Farming Scheme, includes the animal health improvement cycles scheme, where a plan is made with farmers, and they monitor and review it. There was optimism about this scheme as a means to drive change on farms, as it included monetary incentives for implementing those changes. 

#### 2.2.7. The Scotland’s Healthy Animals Website 

Another initiative that was mentioned in Scotland was the Scotland’s Healthy Animals website that was created by the Scottish OH AMU and AMR (SONAAR) team in charge of the publication of the annual SONAAR reports. It was created as a guidance hub for all animal keepers, veterinary and animal health professionals and the public, to provide advice on AMR, biosecurity, and antimicrobial stewardship. A Scottish veterinarian expressed optimism about Scotland’s approach, which focuses on biosecurity and animal health over a focus on hitting antibiotic reduction targets. 

### 2.3. Infection Prevention and Control 

An increase in vaccine use over the last few years was reported by interviewees, representing a shift towards prevention rather than treatment: “*We have increased the vaccines. In some ways we’ve gone from treating for worms to Huskavac on the youngstock. That’s probably quadrupled my vaccine costs*” (Farmer 3). Veterinarians mentioned that it was becoming easier to introduce vaccines to farmers as they were now much more open to vaccination. There was enthusiasm among veterinarians for vaccination as a way to prevent underlying diseases which would otherwise require treatment with antibiotics: “*we’ve looked at our sales in practice of antimicrobials and of vaccines and the antimicrobials have come down and the vaccines have gone up. … I think the antimicrobials were down by maybe 15% and the vaccines were up by 100% or something stupid like that over a period of time. But, yes, there is a lot more BVD vaccination and calf vaccination*” (Vet 2). Various factors were reported to have contributed to this, as illustrated in this quote: “*It is always multifactorial isn’t it. Often somebody seems like they’ve done it because they’ve taken your advice but in actual fact, they are primed to take your advice because they know that their neighbor has done it or they’ve seen an advert. Yes, so a little bit of each*” (Vet 2). The data published by AHDB showed that the total number of cattle vaccines sold in the UK increased by 18% between 2011 and 2022, with the biggest increase in vaccine uptake being for calf pneumonia (45%) and calf enteritis (37%) [34].

Although farmers shared this enthusiasm, the cost of vaccines and labour was mentioned as one of the challenges to vaccination uptake by farmers. One suggestion to address this was to conduct economic analyses of the commonly used vaccines to show their value: “*I think in many cases people are economising and thinking that they’re actually saving a bit of money or finding it’s too labor intensive to fit them in at the correct time. Or maybe have used vaccines in the past and they weren’t correctly targeted, so they were disappointed, so they stopped. And obviously there are cost pressures so that everybody looks down the list of what are the sorts of things we don’t need to do and maybe cross some of them off. So, I think there are three or four reasons there why uptake isn’t higher and probably it would be quite useful if there were costed case studies on some of the common vaccines to actually give people confidence that they might get value for money*” (Vet 4). There are constant trade-offs in decisions for animal-sourced food production which has only become more intense in the current economic climate. The recent demonstrations by some farmers against cheap imports and the worry around new subsidy schemes further highlight the fact that many decisions will be made with economics and livelihood survival in mind. The pressure to continue to deliver food for their nation under more difficult conditions will affect actions taken by livestock producers. If it is not apparent that a vaccine is making any clear contribution then it will not be, or cannot be, prioritised. Therefore, even if costed case studies evidenced the importance of vaccines and the economic benefits, this does not mean this avenue is accessible for all farmers. 

In August 2022, NOAH launched a Livestock Vaccination Guideline for beef, dairy and sheep sectors to enable a best practice approach to vaccination in the livestock sector [35]. In the guideline, vaccinations are divided into two categories. Category one comprises high-priority vaccines such as leptospirosis in dairy and beef cattle, and category two is for vaccines where the use is recommended as best practice depending on farmer and veterinarian review and discussion [35]. This guideline is considered to provide valuable information to farmers and veterinarians to plan tailored vaccination programmes for cattle and sheep. 

When discussing initiatives such as the herd health plan and the AHWP, veterinarians were often positive about the chance this would give them to focus on infection prevention including improvements to housing, nutrition and herd management. They commented that there has been a change in calf feeding which has had a beneficial impact on their immunity and enabled greater resilience to infections, therefore reducing the need for antibiotics. The main health issues reported in calves treated with antibiotics were pneumonia and diarrhea. It was highlighted that in addition to vaccination, there is a need to focus on hygiene and disease prevention to tackle these issues. There were worries among some veterinarians that the elimination of the basic payment scheme would make it more difficult for farmers to keep cows due to the expense, and therefore beef will come from calf-rearers which are less likely to be suckled, and non-suckled calves are more vulnerable to infection and therefore require more antibiotics. The basic payment scheme was a large rural payment scheme providing governmental financial support to the farming sector linked to the amount of land. This system stopped in 2023 and, instead, payments will be made averaging basic payment amounts (from 2020 to 2023) until 2027 when they will be phased out. There are other schemes farmers can apply to, for example, including the AHWP (as previously discussed) and the environmental land management schemes (ELMs) to help with any difference in subsidy received.

Interviewees also mentioned the importance of BVD eradication programmes to lower the burden of this disease and its impact on cattle due to the use of AM to treat this disease and the fact it is highly contagious. Various approaches have been adopted in the different UK nations. In England, the voluntary programme to eliminate BVD from all cattle herds in England (BVDFree) was launched in July 2016 [30,36]. At the end of six years, 6600 herds had registered with BVDFree, representing close to an estimated 49% of the national cattle breeding herd in England. The AHWP released plans to eradicate BVD via a voluntary scheme due to launch in early 2024 [30,32]. In Scotland, the BVD eradication scheme started as a voluntary programme in 2010 and became mandatory in 2013. Since the start of the programme, there has been a continuing downward trend in BVD-infected herds and persistently infected (PI) animals [30,36]. For Wales, in September 2017, Gwaredu BVD (Eradicating BVD) was launched to eradicate BVD from the Welsh national herd. This is a voluntary scheme, which involves BVD screening at the same time as TB screening. At the end of the fourth year of the programme in March 2022, over 83.3% of the 11,000 herds in Wales had been screened. For NI, the NI BVD eradication programme began in March 2016 and important progress has been made to date [30,36]. 

### 2.4. Economic and Labour Concerns 

Many veterinarians raised concerns about the “recruitment crisis” in farm veterinary practice. This affects their ability to spend sufficient time with farmers to fully implement initiatives such as the AHWP annual reviews and to promote efforts to optimise ABU and improve herd health. Veterinarians also noted that it would reduce their availability to complete training: “*There will be a lot of practices that are understaffed and being able to roll out any national schemes will be hard for them. And I think that will have a negative impact in terms of being able to try and roll anything out*” (Vet 1). 

Some participants expressed concerns regarding trade agreements and highlighted the need to ensure that trade agreements do not undermine the goals of the NAP. There were concerns that trade could be permitted with countries that do not take as responsible an approach to AMR as the UK, thereby allowing cheaper food imports from these countries: “*I think there is a little bit of concern in the industry about the trade deals that we’re negotiating and the potential influx of cheaper food imports from other countries*” (Senior Staff). Producers in the UK might be bound to stringent rules that do not apply to products that are coming from other countries. This can be frustrating if they work hard to hit the targets only to find that they are undercut in the market by products that do not meet these targets: “*Well, I know there is probably a bit of scaremongering in the news about it, but I suppose time will tell. But they do make you a bit mad with how they agree to import stuff. It’s a lower standard of what we are allowed to produce at*” (Farmer 6). This frustration is being shown in the farmer demonstrations, mentioned previously. Therefore, these trade agreements apply not only economic pressure on farmers but also moral pressure on the country as there is the possibility of importing meat products with higher AMU than UK-produced meat.

### 2.5. Impact of COVID-19

Farmers did not report an issue with gaining veterinary advice or medication due to the COVID-19 pandemic. Veterinarians agreed that most of their work was not affected by COVID-19, as a lot of it is emergency, essential work, but those activities which were not deemed essential were stopped. This impacted some initiatives such as the BVD Stamp Out initiative. The aim of this programme was to engage 50% of breeding herds in England (dairy and beef) in BVD control by 2021. There was also a slight increase in distance prescribing for non-emergency work, meaning the vets could not assess the true need for antibiotics and this may have led to an increase in unnecessary prescribing, but this was said to have been minimal. 

One of the areas that was impacted by COVID-19 and mentioned by interviewees was vaccination supply. There was a shortage of some of the vaccines, which was frustrating to farmers as they were not able to carry out the booster vaccinations for some diseases. However, there was some suggestion that it was not known if this was an impact due to the UK leaving the European Union or COVID-19. 

### 2.6. Suggestions for Next NAP

With regard to the next revised NAP, several suggestions were made by interviewees. There was an agreement that the focus needs to continue to be on infection prevention and control as this is key to reducing the need to use antimicrobials in the first place. Also, efforts to improve the collection of ABU data and address the obstacles to the uptake of the MH are needed. An example that was mentioned was the need to ensure the compatibility of software between the MH and vet practices: “*So in terms of initiatives going forwards something as simple as medicine hub being able to talk to practice management software*” (Vet 1). 

In addition to AMU data, interviewees highlighted the need to collect structured surveillance data on AMR from veterinary pathogens. This would enable the identification of changes in susceptibility to antibiotics, assess the impacts of interventions and help veterinarians with their clinical decisions: “*I just feel as though there is a big surveillance gap there and certainly, we do very little routine resistance screening. And to me all the effort we go to about responsible antibiotic use it should be in parallel looking for resistance, shouldn’t it, otherwise we’re doing it all without an evidence base, we don’t actually know what’s getting better, what’s getting worse, where things are getting worse…. I’d say nearly all the resistance data that we get as a practice we get because we take part in research projects, and so if we didn’t, then we would have virtually no resistance data at all from the pathogens and the bacteria that, you know, are on our client farms*” (Vet 8).

Regarding vaccines, there were issues with the supply of some of the vaccines. It was highlighted that it was crucial to ensure that vaccine supply is adequate and that farmers can access the vaccines when they need them: “*We need to make sure that vaccine supply is available. More broadly, we need to make sure that UK farmers still have the widest access to veterinary products that they can, and that be it Covid or Brexit or any other trade deal, doesn’t limit those—that supply. Because that would put us at a disadvantage to other competitors potentially*” (Senior Staff).

Another suggestion emphasized the need for pen-side testing at the farm level. Pen-side testing, also known as point-of-care testing, was seen to be valuable for farmers and veterinarians to make informed decisions and address health issues in a timely manner, thus contributing to more responsible use of antibiotics. 

## 3. Discussion

This study aimed to explore the progress made in the beef sub-sector in addressing specific commitments in the UK AMR NAP 2019–2024 related to improving animal health and welfare and responsible AMU. The findings show that progress has been made in addressing NAP commitments with various initiatives implemented including the MH, the WLBP AMU calculator, training for veterinarians and farmers, and the AHWP. These various initiatives have contributed to an increase in awareness of AMR, strengthening of the relationships and engagement between farmers and veterinarians, improvement in antibiotic use practices, and greater vaccination uptake. However, this study also highlighted challenges and barriers that need to be addressed in order to assess the impacts of the initiatives implemented. 

Initial feedback from interviewees on the activities implemented has been positive, but long-term data on AMU and AMR and intervention efforts are needed to be able to evidence their impacts over time and attribute the changes observed to the various activities. In the latest UK-VARSS report, a mg/PCU figure could not be provided yet for beef cattle, thereby preventing benchmarking and comparison with other livestock [23]. A figure was published in the latest RUMA TTF report based on 5% of the beef population submitted to the MH, using the CHAWG methodology [27]. Many challenges to the uptake of the MH were reported including the incompatibility of software between the MH and veterinarians, which makes submitting the data an extremely lengthy process. Although all veterinarians were aware of the MH, only one farmer had knowledge of it due to their involvement in the TTF. This might be due to the system not being fully operational yet. Even when it is fully operational, there might be challenges due to the heterogeneous nature of the beef sub-sector, which makes unified messaging a challenge. In general, sectors with fewer producers tend to find it easier to communicate and make improvements in antibiotic stewardship and data capture [15]. Conversely, as the number of producers within the sector increases, it becomes more challenging to drive change as it becomes harder to engage the remaining ones [15]. For example, the integrated nature of the poultry sector was an important factor in helping it to collect AMU data [14]. Interviewees highlighted the importance of providing support to the cattle and sheep industry to address the barriers to the MH, including the allocation of necessary funding. One suggestion made was to focus at the initial stage on collecting veterinary practice level data as a proxy for the quantity of antibiotics used, but this will require dedicated funding for veterinary practices to facilitate the data input as it is a time-consuming and competes with other veterinary practices priorities. Additionally, it is important that veterinarians record treatments in their practices by species rather than at the farm level, in order to obtain accurate information on AMU for each species. The MH platform, once sufficiently populated, will provide a representative overview, allow monitoring of AMU trends in the cattle and sheep sectors, inform the implementation of appropriate management strategies, and permit assessment of the impacts of interventions.

The importance of collecting structured surveillance data on AMR from veterinary pathogens was also highlighted. Currently, these data are collected as part of the clinical surveillance programme for AMR in food-producing animals in the UK, which is based on passive surveillance with samples from carcasses and other diagnostic samples submitted by farmers or their veterinarians to government laboratories [28]. Given that representativeness is not known, results cannot be extrapolated to the whole livestock population. Moreover, the levels of resistance in these isolates might be higher than those in the wider population, since treatment failure is a frequent reason for submission of samples [28]. Therefore, there is a need to develop a structured surveillance programme for AMR from veterinary pathogens that would enable the identification of changes in susceptibility to antibiotics over time. The information generated would be crucial to investigate the links between AMU in animals and the development of AMR, to support antimicrobial stewardship initiatives such as the development of veterinary antibiotic treatment guidelines, and to assess the impacts of interventions [37,38]. At the European level, there is a call to develop a European antimicrobial resistance surveillance network in veterinary medicine (EARS-Vet) to report on the AMR situation, follow AMR trends and detect emerging resistance in selected bacterial pathogens in animals [37,39]. Informants also emphasised the importance of developing pen-side diagnostic tests. These resources should aid farmers and veterinarians in making well-informed decisions directly on the farm, enabling them to address health issues promptly. Consequently, this should contribute to a more responsible approach to antibiotic usage.

Although veterinarians and farmers had little awareness of the NAP, they were all aware of the issue of AMR, the need for responsible antibiotic use, and RUMA TTF work. The various initiatives implemented have contributed to an improvement in antibiotic use practices, but these efforts are built on prior work by the UK government to tackle the issue of AMR. In 2014, the then Prime Minister commissioned an independent review of AMR led by the economist Lord Jim O’Neill [10]. The final report, published in 2016, offered ten recommendations to address the problem [10]. The review was influential both nationally and internationally, and the UK government published a formal response setting a series of key commitments it would make [40]. RUMA TTF was formed in 2016 to respond to the AMR review, and it continues to play a key role in antibiotic stewardship [22,30]. RUMA TTF takes into consideration NAP commitments when developing the sectors’ specific targets, and having a representative from the VMD in the TTF helps to ensure that the knowledge of the NAP is fed into the discussions. Additionally, the British Veterinary Association (BVA) attends RUMA TTF as an observer and provides inputs to the targets. Consequently, on-the-ground practices align with the NAP’s objectives and relevant information on good practices reaches the farmers even with little awareness of the NAP. Increasing awareness of the NAP among farmers and veterinarians may help them to see the big picture and national efforts going on to tackle the problem. This knowledge might be a motivating influence, as farmers may recognise how their actions can have wider societal benefits. This awareness can be enhanced by incorporating NAP information into training programmes, and disseminating it through industry bodies such as RUMA and BVA.

A very important but insufficiently recognised outcome of all the initiatives was the time and opportunity they gave to help build and strengthen the farmer–vet bond. Initiatives that strengthen the relationship between farmers and veterinarians were considered beneficial for building trust. Furthermore, the importance of having time to discuss relevant issues was emphasised. This extra time enables vets to learn more about issues on the farm, so that the vet and farmer can identify the most impactful actions collaboratively, ultimately contributing to improved farm management and increased awareness of appropriate AMU. This is in line with a previous study, which highlighted that beef farmers value the advice from their veterinarians and would like more information on disease control in their herds to support the reduction in AMU [25]. This relationship is the foundation of any initiatives and/or movement towards transformative and sustained change in the system. Some of the initiatives implemented that were considered to provide a good opportunity to improve engagement between veterinarians and farmers were the AHWP, the herd health plan, and the AVC and FVC programmes. Having a good farmer–veterinarian relationship can aid herd health plans to be more effective and for farmers to see the value of these plans to them. The bi-directional learning process allows veterinarians to gain deeper insights into the farms and farmers to receive valuable tailored advice from their veterinarians. Interviewees mentioned that it was still early to comment on the impacts of the AHWP, but there was optimism regarding the initiative’s potential. The development of bespoke herd health plans collaboratively between farmers and veterinarians, focusing on health and welfare and responsible use of antibiotics, and subsequently implementing the necessary changes, was perceived to be an effective strategy for reducing AMU. The AVC programme had good coverage with at least one VPC from nearly 90% of veterinary practices in Wales. For the FVC programme, in September 2023, there were 892 FVCs across the UK, 44 SMART goals were set and 18 teams had been created [27]. Although veterinarians acknowledged the usefulness of these programmes, they expressed concerns that they could easily be missed due to time constraints and competing demands at their practices. An evaluation of these programmes would allow an understanding of the challenges to their implementation and whether there have been any changes in prescribing practices. 

An increase in vaccination uptake was reported by interviewees, which was seen as a positive change that needs to be sustained. Various factors were considered to contribute to this increase, including advice from veterinarians, the influence of neighbouring farms, and messages from adverts. The data published by AHDB showed that the total number of cattle vaccines sold increased by 18% between 2011 and 2022, with the biggest increase in vaccine uptake being for calf pneumonia (45%) and calf enteritis (37%) [34]. However, because of COVID-19, there was a disruption in the vaccine supply and farmers had to skip some of the vaccines. This was perceived as a factor that could discourage the uptake of vaccination by farmers and therefore could hinder future progress on NAP commitments to encourage greater uptake of vaccines. Hence, it is important to ensure that there is adequate vaccine supply. 

While several interviewees considered beef farmers to be low users of antibiotics, they expressed concerns regarding AMU in calf-rearing units. Calf-rearers raise young animals predominantly from the dairy sector [15]. Mixing calves from different sites, combined with stress from transport increases the risk of disease transmission at a time when calves are more susceptible to disease [25]. An increase in the use of dairy calves for beef production has been reported in the UK due to the commitments to eliminate euthanasia of calves [26]. This is a positive change to avoid waste in the system, but this overlap between the two sub-sectors can create challenges in terms of disease mitigation and interventions to reduce AMU. Young animals are vulnerable, and the care received during the initial days of their lives can have a big influence on their subsequent susceptibility to disease. Therefore, measures implemented in the beef sub-sector need to take into account this link. 

Having sufficient funding is a key driver for initiatives to be successful. Therefore, it is imperative to ensure sustainable change that would lead to sustainable practices. Given the financial challenges farmers are encountering, ensuring adequate funding becomes pivotal in mitigating some of these challenges and encouraging compliance with any proposed interventions. Additionally, farmers have expressed concerns about potential trade deals with countries with lower standards than the UK, which could compromise their efforts to uphold high-quality production standards. Hence, it is important to ensure that trade agreements do not undermine the objectives of the NAP.

The updated UK NAP “Confronting antimicrobial resistance 2024 to 2029” was published in May 2024. It focuses on nine strategic outcomes organised under four themes: (1) reducing the need for, and unintentional exposure to antimicrobials; (2) optimising the use of antimicrobials; (3) investing in innovation, supply, and access; and (4) being a good global partner [41]. The new NAP built on the achievements and lessons from the first NAP including the outcomes of the evaluation of the UK AMR NAP 2019–2024 that this study is part of.

## 4. Methods 

### 4.1. General Overview 

A theory of change (ToC) was developed to gain an overview of the different actions and interventions implemented to improve health and welfare in the beef sub-sector and responsible antimicrobial use (AMU). This ToC was used to gain an initial understanding of change pathways that would lead to the reduction in AMU (and consequently AMR) and was used to develop a topic guide for semi-structured interviews with stakeholders related to the beef sub-sector. A total of 21 stakeholders were interviewed to understand experiences and progress made towards the achievement of NAP commitments.

### 4.2. Theory of Change 

**Figure 1:**
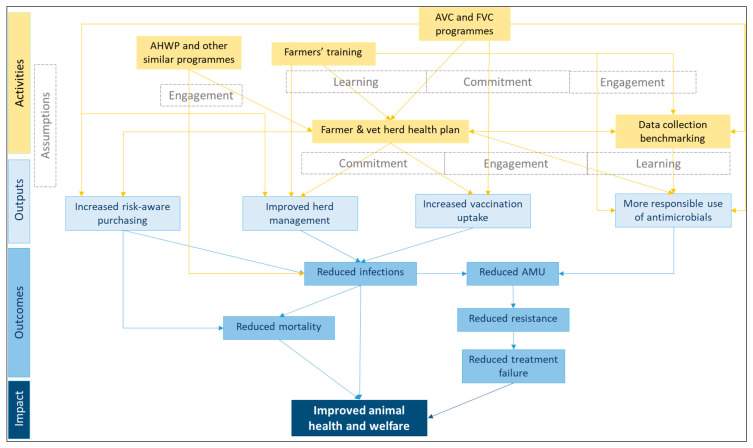
Graphical presentation of the theory of change presenting the activities to improve health and welfare and responsible AMU in the beef sub-sector with their underlying assumptions (in grey), outputs, outcomes, and impact.

The ToC was developed by the authors from a desk review of publicly available reports [11,15,22,30] to understand the intention and possible change mechanisms of the proposed and ongoing activities to improve health and welfare in the beef cattle sub-sector and responsible AMU and to have a framework that would guide the development of questions and the subsequent analysis. It describes change pathways through which interventions or programmes can produce outputs and outcomes; in this case, health- and AMU/AMR- related ones (Figure 1). 

Acronyms: AVC: Arwain Vet Cymru; FVC: Farm Vet Champions; AHWP: Animal Health and Welfare Pathway; AMU: Antimicrobial Use

- Activities: With the UK AMR NAP 2019–2024, there was a continuation of the collaborative work between the VMD and the livestock sectors. The NAP-related activities elaborated by the livestock sectors (relevant to beef cattle) focus on the main areas listed below [11,15,24,30]. The key areas in bold are those mentioned in the RUMA TTF 2020 report, and the details afterwards comprise a mixture of their descriptions and authors’ interpretations of how the activities are intended to have their effect.
- Data collection: The collection of ABU data would allow an understanding of which products were being used to treat which animals and exploring where opportunities might exist for potential improvements in product use. In addition, it was expected that knowing how much antibiotic they use would allow farmers to set use targets with their veterinarians. They could also compare their use with other users in the field and use this as an incentive for change. These factors would likely lead them to be more engaged and committed to implementing appropriate actions.
- Veterinarian/farmer engagement: Enhancing veterinarian and farmer engagement would allow for building trust and encouraging more health planning and preventative approaches to disease control, which would ultimately lead to reduced infections and improved animal health and welfare.
- Herd health planning: This element relies on the veterinarian and farmer developing herd health plans and reviewing health and performance indicators annually. The herd health plan focuses on areas including upload and benchmark of ABU data, HP-CIAs use or any routine prophylaxis, patterns of persistently high use, disease prevention, risk-aware purchasing, and health and welfare outcome metrics such as reduced mortality.

These activities are expected to lead to more responsible AMU, improvements in herd management, greater use of vaccinations and risk-aware purchasing that can reduce infections and AMU, thereby contributing to reduced resistance, and improved animal health and welfare, as illustrated in the blue boxes in Figure 1. This ToC is based on key assumptions related to learning, engagement, and commitment. Firstly, learning is seen as important in providing veterinarians and farmers with up-to-date information and training on best practices to enhance their knowledge and skills. Secondly, engagement between the various stakeholders is deemed necessary to create shared responsibility, vision and approach. Finally, sustained improvements in the beef sub-sector are estimated to require a long-term commitment from everyone involved.

### 4.3. Semi-Structured Interviews 

A qualitative approach using semi-structured interviews was employed to explore the experiences and views of stakeholders on the progress made in the beef sub-sector in addressing NAP commitments and achieving change, and any suggestions they might have for the next NAP. Interviews were conducted using an interview guide developed by the evaluation team (see File S1). It contained questions on; (1) respondents’ roles and their experience with the implementation of the NAP, (2) perceived effects of the NAP on ruminant sectors and in particular the beef sub-sector, (3) examples of initiatives that changed due to the NAP, (4) benefits, challenges, and responses, (5) motivation for, and satisfaction with, changes, (6) effects of the COVID-19 pandemic, (7) effects of the initiatives on local implementation, (8) influence of the NAP on different stakeholders, (9) lessons learned and future plans, and (10) desired changes to the NAP. 

A total of 21 semi-structured interviews were conducted with representatives from government organisations, farms, veterinary practices, levy boards and livestock associations. Purposive sampling was used to select informants who were based in organisations involved directly or indirectly in livestock production in general and beef production in particular, and antimicrobial use policy and regulation. Interviews were conducted by HB and LW between January and July 2023 and lasted for up to 60 min. Fifteen interviews were conducted remotely and six interviews with farmers were conducted in person at farms. Information on the roles of participants is given in Table 2. 

All interviews were recorded and subsequently transcribed verbatim by a professional company. The transcripts were checked for accuracy by HB and then transferred into NVivo14 for data management and analysis. A thematic analysis following Braun and Clarke’s approach was used [42]. The following steps were followed: familiarisation, coding, generation of initial themes, development and reviewing of themes, refining and naming themes and writing up [43]. Coding was undertaken using an initial coding framework based on the topic guide and adapted through multiple rounds of re-coding. A combined approach to the analysis was used enabling themes to be developed both deductively from the topic guide and inductively from the experiences and views of participants. Key themes identified were used as headings to organise the findings. Summarized thematic information from all participants, as well as the relevant literature, were included. Direct quotes from the transcripts were used to illustrate key points. 

### 4.4. Ethics and Consent 

The study received ethical approval from the London School of Hygiene and Tropical Medicine (Reference number 27930) and the Royal Veterinary College (RVC Social Science Ethical Review Board reference number: URN SR2022—0151).

An information sheet with the description of the project and the objectives of the interview was sent to participants prior to the meeting and they had the possibility to clarify any questions before the interview. Written consent for participation in the interview and an audio recording were obtained from participants before the interview. 

## 5. Conclusions

This study showed that various initiatives have been implemented in the beef sub-sector to address AMR NAP commitments, but their impacts have not yet been measured and remain open to further research. For the next phase of the NAP, the following should be considered:

- A continued focus on the enhancement of data availability in this sub-sector.
- A careful examination of resource availability to ensure the sustainability of effective initiatives including the consideration of competing demands on people’s time.
- Consider how surveillance infrastructure, medical supply chains, veterinary service provision and trade agreements can support and maintain the success of the efforts in the longer term.
- Build on the human relations within the system, particularly the farmer–vet bond, bidirectional learning and co-design, thereby creating greater trust, mutual respect and a common language that are fundamental for sustainable effective change in relation to the desired outcomes of the AUK MR NAP 2024–2029.

## Figures and Tables

**Table 1:** NAP commitments and initiatives to address them.

NAP Commitments	Initiatives
Implement plans with the veterinary profession and livestock industry to improve animal health and address endemic disease issues through disease control schemes, veterinary advice and health planning, and tools for promoting knowledge transfer (such as guidance, training and communication).	Herd health plan, Animal Health and Welfare Pathway (AHWP), Arwain Vet Cymru (AVC), Farm Vet Champions (FVC), NOAH Livestock Vaccination Guidelines for cattle and sheep sectors
Incentivise regular monitored animal health and welfare reviews as a key strategy for infection prevention and control in farmed animal enterprises. Monitor the effectiveness of animal health and welfare interventions to learn and disseminate best practices at regional and national levels.	AHWP
Encourage greater uptake of available vaccines	Herd health plan, AVC, FVC, NOAH Livestock Vaccination Guidelines for cattle and sheep sectors
Work with industry and the veterinary profession to improve our understanding of available disease data. Consider how to expand this and share at farm, regional and species levels as well as nationally. Use in tandem each country’s multispecies databases to improve disease surveillance and reduce antibiotic use.	Medicine Hub (MH), Welsh Lamb and Beef Producers Ltd. (WLBP) (Aberystwyth, UK) AMU calculator
Work collaboratively across, for example, UK government administrations, the veterinary profession and agriculture industry to develop appropriate training, guidance and other communications for those who are (or influence or are training to be) antimicrobial users and prescribers to encourage the uptake of recommended practices. And evaluate their impact.	AVC, FVC, other training for veterinarians and farmers, and Scotland’s Healthy Animals website
Improve the accuracy, availability and coverage across the UK of antibiotic use data in the main livestock sectors. This will include working collaboratively across UK government administrations, the veterinary profession and agriculture industry to implement sector targets by the end of 2024.	MH, WLBP AMU calculator

**Table 2:** Participants’ organisations and roles.

Organisation	Role of Interviewee
Veterinary Medicines Directorate (VMD)	Senior Officer
Welsh Government	Senior Officer
Scottish Government	Veterinary advisor
Responsible Use of Agriculture Alliance (RUMA)	Senior Staff
Agriculture and Horticulture Development Board (AHDB)	Senior Staff
Private veterinary practice	Veterinarians (Vet 1 to Vet 7)
Private veterinary practice	Veterinarians with roles in RUMA Targets Task Force, British Cattle Veterinary Association (BCVA), and FVCs (Vet 8 to Vet 10)
Beef farms	Beef farmers (Farmer 1 to Farmer 6)

## Data Availability

The datasets generated and analysed during the study (interview transcripts) are not publicly available due to the non-anonymised nature of the data.

## Supplementary Materials

The following supporting information can be downloaded at: https://www.mdpi.com/article/10.3390/antibiotics13090839/s1, File S1: Topic guide for the interviews.
