# Supplementary material for: Progress and Challenges: Implementation of the UK Antimicrobial Resistance National Action Plan 2019–2024 within the Beef Cattle Sub-Sector
Source: Antibiotics (Basel). 2024 Sep 4;13(9):839. doi: 10.3390/antibiotics13090839 (PMC11428892; doi:10.3390/antibiotics13090839)
Supplement: Supplementary file 1 [file antibiotics-13-00839-s001.zip › antibiotics-3138259-supplementary.pdf]

<u>Topic Guide for the interviews:</u>

Introduction and consent

<u>Questions to veterinarians</u>

1. What are your main concerns in regard to AMR?
    a. If they don't mention beef, prompt them
2. What are your thoughts about using AMs in cattle, in particular in beef animals?

3. Are you aware of the UK AMR NAP?

4. Any initiatives to improve the health and welfare of ruminant animals? Any particular actions in the beef sub-sector?
    i. Grants to farmers as part of the AHWP for an Annual health review with their vets and to apply for equipment and infrastructure that would improve the health and welfare of the animals.
    ii. Herd health plan (what changing in particular),
    iii. Increased vaccination (what vaccinations and why)
    iv. Training of vets and farmers
    v. More engagement with the farmers

5. Can you describe how the AMR NAP affected your work on addressing AMR? Can you give examples of initiatives to address AMR in your area of work that have been put in place over the last three years?
    a. Any initiatives that have started, stopped or changed direction? Why did this happen? Who was driving these initiatives? Who were these initiatives directed at?
    Prompts:
        i. Antimicrobial stewardship activities such as the FVC and AVC (welsh one) programmes
        ii. Antibiotic usage data collection and benchmarking using the Medicine Hub (started 2022)
6. What is your opinion about these initiatives?
    a. Do you think they made a difference (or they will make a difference if the activities have just been implemented)? How? If not, what else do you think would make a difference?
    b. What have been the impacts of these initiatives? (e.g. Reduced infections, reduced antimicrobial use, reduced mortality, reduced treatment failure, improvement in cattle health, improvement in recording in the practice) How was this assessed? Did you see any changes in farmer attitude, knowledge, practices. Or yourself/colleagues? No measurable benefits.
7. Can you describe any challenges with implementing these initiatives? How have they been addressed?
    a. Any impacts of Covid-19 pandemic?

8. Can you describe any changes in how you prescribed antibiotics over the last three years? Why did these changes happen?
9. What is your opinion about the FVC programme (or AVC)?
   - Did you participate to this programme? If yes, ask questions below.
   - If no, why not? Do you know about it? Are you planning to participate?
10. How did you find the learning modules of the FVC (or AVC) programme?
    - Which parts were particularly important? Where they useful? In what way? Did you learn anything new that you didn't know before?
11. Can you describe any impacts of the FVC (or AVC)?
    - Any changes in your practice as a result of the FVC (or AVC)? Any changes in your prescribing or recording of antimicrobials?
    - Any changes to your relationship with farmers?
12. Do you know about FVC SMART goals? What is your opinion about the FVC SMART goals?
    - Are you using it or planning to use it? can you describe your experience?
13. What are your views on the reported shortage of vets, particularly farm vets? Do you think this could have an impact on the efforts to tackle AMR? In what way?

Next phase:

14. Can you describe any changes that you would like to see in the next NAP? Relevant to include?

Questions to beef farmers

1. Are you aware of the UK AMR NAP?
2. Have you got any concerns about using antibiotics in beef and the development of resistance in animals and humans?
   a. How can these be addressed? (let them expand if the option is there)
3. What are your thoughts about using AMs in beef animals?
4. Can you describe any changes in how you used antibiotics over the last three years?
   a. Why did these changes happen?
5. Can you describe any initiatives to address AMR that have been put in place in the last three years?
   a. Any initiatives to improve the health and welfare of the animals on your farm?
   b. Any initiatives that have started, stopped or changed direction? Why did you do it? How did you find about it?
      i. Antibiotic usage data collection and benchmarking using the MH
      ii. Grants to farmers as part of the AHWP for an Annual health review with their vets and to apply for equipment and infrastructure that would improve the health and welfare of the animals
      iii. Red tractor medicine training?
      iv. Herd health plan
      v. Increased vaccination
      vi. Improvement in farm management
      vii. Training

   c. Any changes in the management of animal infections in your farm? Why did these changes occur? (Prompts: Who influences these changes? (the influence of the NAP), What farmers do and why? What infections are? How are they perceived as a problem)
6. What is your opinion about these initiatives?
   a. Do you think they made a difference (or they will make a difference if the activities have just been implemented)? How? If not, what else do you think would make a difference?
   b. What have been the impacts of these initiatives? e.g. Reduced infections, reduced antimicrobial use, reduced mortality, reduced treatment failure, improvement in AB recording
7. Can you describe any challenges with implementing these initiatives? How have they been addressed?
   a. Any impacts of Covid-19 pandemic?
8. Can you describe how you record antibiotic usage on your farm? And general animal health. So do they know if levels of infections are going up and down? Or missing hidden diseases?
   a. Do you upload usage data to the Medicine Hub? If yes, what is your opinion about using the MH? If no, why not?
9. What are your views on accessing veterinary services for beef farmers and the quality of these services?
   a. Any shortage of farm vets?
10. Have there been any changes in your relationship with your vet in the last few years?
    a. If yes, what are those changes? Why do you think they occurred?
11. Do you always get your antimicrobials from your vet? Or do you purchase them from other sources?
    a. If so where and how do you know what to buy?

Next phase:

12. Can you describe any changes you would like to see in the next NAP?

Questions for stakeholders from government organisations in the four UK nations and industry organisations

Your role in the implementation of the NAP

1. Can you describe your role and how it relates to the implementation of the UK AMR NAP?

Implementation of the NAP with a focus on the ruminant sector in general and the beef sub-sector in particular

2. Can you describe how the NAP affected your work on addressing AMR in the ruminant sector in general and beef sub-sector in particular? Can you give examples of initiatives that have changed because of the NAP?

- Any initiatives that have started, stopped or changed direction? Why did that happen? (E.g. Farm Vet Champions-FVC, Arwain Vet Cymru-AVC, Animal Health and Welfare Pathway-AHWP, Farmers' trainings)
- Who were these initiatives directed at and how did they change?
- Do you think there is anything that would not have been done if the NAP hadn't been there?

3. Do you report progress on those initiatives? If yes how, and what happens as a result of that?
    - Do you have any specific reports? What happens with these reports? Did they make any difference? If yes, how did you find out?
4. Did you perceive any benefits or added value arising from these initiatives? What are they? How they were assessed, documented or described?
5. Can you describe any challenges with implementing these initiatives? How have they been addressed?
    - Competing priorities and limited resources?
    - Knowing how to implement key activities?
    - Working across the four UK nations?
    - Working at local and national level?
    - Working across disciplines or sectors?
6. Has the implementation of the NAP changed in response to the management of the COVID-19 pandemic? If yes, how?

Effect on local implementation

7. How do measures/initiatives transfer to local implementation?
8. How do you anticipate that the measures/initiatives (those already occurring or those anticipated to occur) might change practice at local level? Do you already see some changes occurring? In what way?
    - Who did you influence with that work? In what way? (This might be people changing things or it can be people affected by the change)
9. Can you describe any AMR-relevant work that you do with local veterinarians/farmers/other stakeholders at local level to support the implementation of the NAP?
    - What have been the challenges? How were they addressed?
10. Thinking about the work on the implementation of the NAP that you have done locally, is there anything that you would do differently in the future, and why?
11. What has worked well and could be used in other areas as well?

Next phase

12. Can you describe any changes you would like to see in the next phase of the NAP?
    - Any changes in the process of the implementation of the NAP, governance, collaboration, data use?
